# Facilitators and barriers of implementing the chronic care model in primary care: a systematic review

**DOI:** 10.1186/s12875-014-0219-0

**Published:** 2015-02-06

**Authors:** Mudathira K Kadu, Paul Stolee

**Affiliations:** School of Public Health and Health Systems, University of Waterloo, 200 University Ave W, Waterloo, Ontario N2L 3G1 Canada

**Keywords:** Chronic care model, Chronic diseases, Primary care, Quality improvement, Intervention implementation, Organizational change

## Abstract

**Background:**

The Chronic Care Model (CCM) is a framework developed to redesign care delivery for individuals living with chronic diseases in primary care. The CCM and its various components have been widely adopted and evaluated, however, little is known about different primary care experiences with its implementation, and the factors that influence its successful uptake. The purpose of this review is to synthesize findings of studies that implemented the CCM in primary care, in order to identify facilitators and barriers encountered during implementation.

**Methods:**

This study identified English-language, peer-reviewed research articles, describing the CCM in primary care settings. Searches were performed in three data bases: Web of Knowledge, Pubmed and Scopus. Article abstracts and titles were read based on whether they met the following inclusion criteria: 1) studies published after 2003 that described or evaluated the implementation of the CCM; 2) the care setting was primary care; 3) the target population of the study was adults over the age of 18 with chronic conditions. Studies were categorized by reference, study design and methods, participants and setting, study objective, CCM components used, and description of the intervention. The next stage of data abstraction involved qualitative analysis of cited barriers and facilitators using the Consolidating Framework for Research Implementation.

**Results:**

This review identified barriers and facilitators of implementation across various primary care settings in 22 studies. The major emerging themes were those related to the inner setting of the organization, the process of implementation and characteristics of the individual healthcare providers. These included: organizational culture, its structural characteristics, networks and communication, implementation climate and readiness, presence of supportive leadership, and provider attitudes and beliefs.

**Conclusions:**

These findings highlight the importance of assessing organizational capacity and needs prior to and during the implementation of the CCM, as well as gaining a better understanding of health care providers’ and organizational perspective.

## Background

The prevalence of chronic diseases is globally on the rise, with cardiovascular diseases, respiratory disease, diabetes, cancer, and other chronic illnesses being major contributors to disability [1,2]. In Canada, two out of five people have at least one chronic disease. Chronic disease is a major driver of health care expenditure, reaching approximately $68 billion in Canada in 2010 [3]. The current health care system is oriented towards episodic and acute care, making it unprepared to address the multi-faceted and complex needs of those with chronic diseases [4,5]. Given the need for continuity, comprehensiveness and coordination, primary care has been suggested as potentially playing a central role in effective management and integration of care [6]. However, literature on current practice suggests that patients often receive inadequate care, with limited physician involvement in disease management, and little coordination and communication among care providers [7].

In response to these challenges and the call for redesigning care delivery for chronic diseases, Wagner and colleagues developed the Chronic Care Model (CCM) [8,9]. The CCM was developed to bridge the gap and translate knowledge between evidence-based chronic disease care and actual care practices. The framework which is centered in primary care, ‘conceptualizes care as prepared practice teams in productive interactions with informed, activated patients’ [10]. It posits six interrelated elements that are key to high quality chronic disease care: self-management support, redesigning delivery systems, decision support that is system wide, clinical information technology, linkages to community resources, and health care system organization [10,11]. The components seek organizational change at the systems’ level, promoting and delivering care that is evidence-based through using clinical tools such as guidelines, utilizing information systems that improve patient data sharing across the organization and between providers, engaging and empowering patients in their care, and mobilizing community resources to meet patient needs [11]. The CCM and its various components have been widely adopted and evaluated, with results showing that it improves patient care and clinical outcomes, and reduces care utilization and costs [12-16].

Despite the extensive evaluation of quality improvement (QI) initiatives, and research on CCM-based interventions, particularly across the United States, little is known about different primary care experiences with its implementation, and the factors that influence its successful uptake [10,14,17,18]. The model provides no clear blueprint on how each component can be implemented in practice, and there is considerable heterogeneity in the types of interventions implemented in primary care in support of the CCM [10]. Previous reviews synthesizing evidence on the CCM have focused on associated care changes, clinical outcomes and cost-effectiveness [10,14,19]. Although a recent systematic review by Holm and Severinsson identified barriers and facilitators of successful CCM implementation in primary care, it was specific to depression management in the US [20]. An understanding of the barriers and facilitators of implementing the CCM, in different care settings is important for several reasons. A barrier in this context is defined as any factor that hinders or impedes care change processes of CCM implementation. First, there are numerous contextual factors that enable organizational change and successful translation of evidence into practice [21,22]. Some of the factors previously identified include: evidence fit and relevance to the organizational context, staff relationships and collaboration, availability of resources, strong and committed leadership, and a culture supportive of change [22-24]. Second, given the complex and multifaceted nature of the model, primary care organizations can face difficulties with its implementation [12]. This is particularly the case given that there are no guidelines available on how to effectively operationalize CCM elements across different settings [25]. We therefore aimed to identify and review evidence on the challenges and barriers encountered while implementing the CCM in primary care.

## Methods

We conducted a systematic literature review to synthesize findings of studies that implemented the CCM in primary care, in order to identify facilitators and barriers encountered during implementation. Barriers and facilitators were interpreted using the Consolidated Framework for Implementation Research (CFIR) [26]. As this research did not involve human subjects, we did not seek ethics clearance for the project.

### Data sources

This study identified English-language, peer-reviewed research articles, describing the CCM in primary care settings. Searches were performed in three data bases: Web of Knowledge, PubMed and Scopus. These databases include Medline, EMBASE and the National Library of Medicine. The PubMed and Scopus search strategy used the following MeSH terms to describe ‘primary care’: primary health care, general practice and family practice. Since there were no MeSH terms for Chronic Care Model, the term was put under quotation marks during the search. In order to ensure a comprehensive search that included all studies that implemented the CCM, MeSH terms for ‘implementation’ were not used in the search. This strategy was also used to avoid excluding studies that might not have identified the term in their titles and abstracts. Search terms and concepts were combined using the Boolean and Proximity operator ‘OR’, while concepts were combined using ‘AND’ and ‘Near’ (Table 1).

**Table 1:** **Key words used in search strategies**

Concept*	Relevant key words**
Primary Health Care	Care, Primary Health; Health Care, Primary; Primary Care; Care, Primary; Primary Healthcare, Healthcare, Primary
General Practice	General Practice
Family Practice	Family Practices; Practice, Family; Practices, Family
Chronic Care Model	‘Chronic Care Model’

*Concepts were combined using the Boolean & Proximity operators AND or NEAR (as databases allow) and the search terms within each concept were combined with OR.

**Keywords were searched using truncation and phrase symbols when appropriate to ensure precise and comprehensive results.

A second strategy adapted from Coleman and colleagues involved searching articles from Web of Knowledge Science Citation Index, which cited the five foundational CCM articles by Wagner and colleagues and Bodenheimer and colleagues [8-10,14,27,28].

In addition, hand searching of the reference lists in all articles that met the inclusion criteria outlined below was performed to identify any missed relevant articles. Search terms used in both search strategies are described in Table 1.

### Study selection

**Figure 1:**
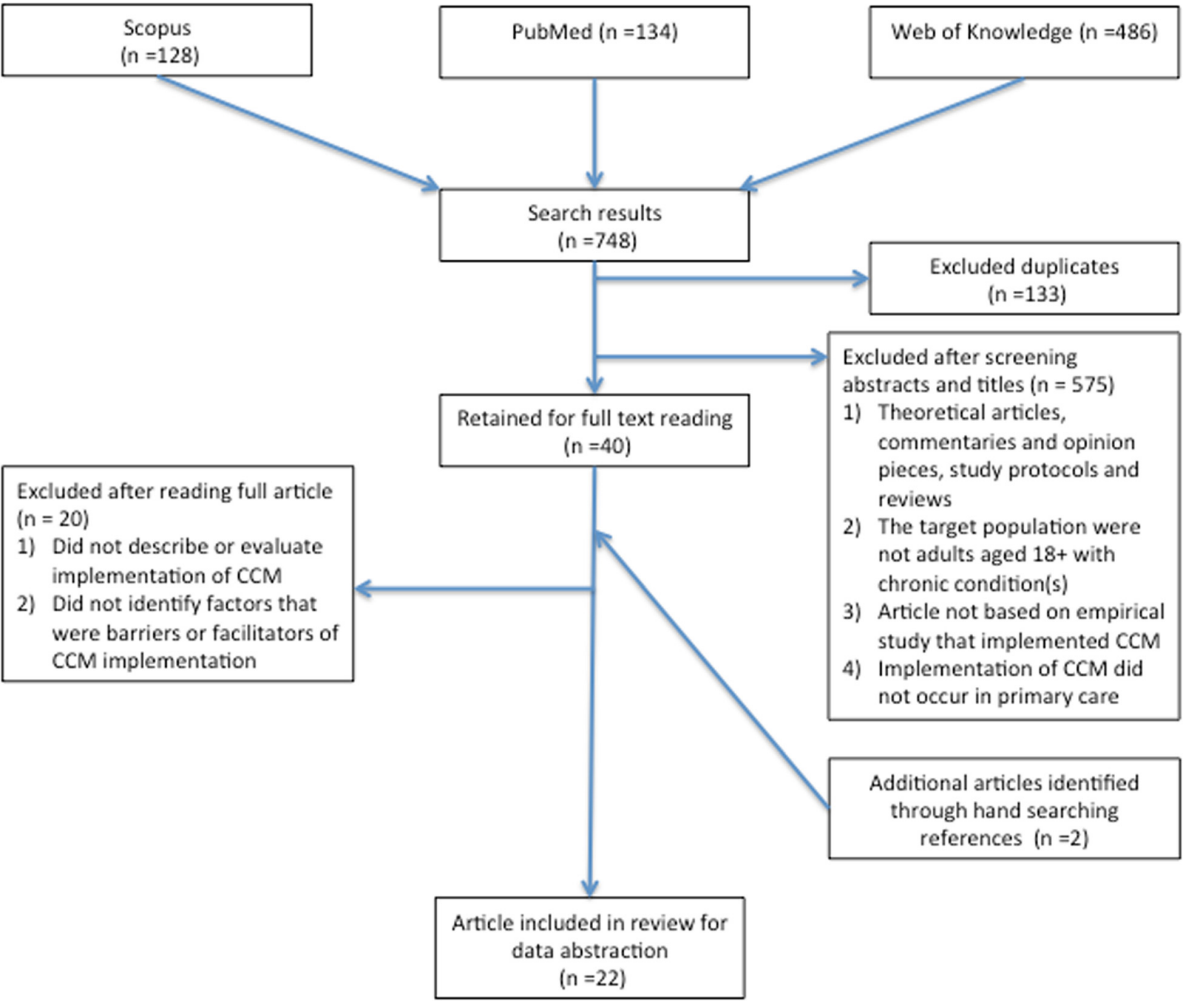
**Exclusion and inclusion criteria for article selection.**

Citations were downloaded and screened in Refworks, an online citation manager tool. Article abstracts and titles were read based on the exclusion and inclusion criteria detailed below. If the reviewer could not determine whether to exclude an article based on its abstracts and title, then it was retrieved for full text reading. Figure 1 displays the process involved in study selection.

Exclusion criteria:

1. Articles published before 2003 and in languages other than English; this year was chosen as the search cut-off to follow the publication date of the last CCM foundational paper by Bodenheimer and colleagues [10], thus reflecting studies that implemented a more mature conceptualization of the model
2. Articles that solely described the CCM conceptually, i.e., did not report on an actual implementation of the model, commentaries and opinion pieces, study protocols, reviews including: systematic and narrative reviews, and meta-analyses
3. The target population of the study was not adults aged 18+ with chronic conditions
4. Articles arguing or providing the rationale for implementation of CCM in primary care, but which were not based on empirical studies.

Inclusion criteria:

1. Articles describing or evaluating the implementation of the CCM. Implementation had to refer to efforts which used change strategies to promote use of evidence-based practices or programs [29]
2. Implementation of the CCM had to occur in primary care, which is defined as integrated and accessible healthcare, delivered in the context of family and community [30].
3. Articles identifying barriers and/or facilitators of CCM implementation.

### Data abstraction

The methods used for the study selection and data abstraction in this systematic review are aligned with those in the PRISMA statement. The PRISMA statement provides an evidence-based checklist intended to improve the standards of reporting in systematic reviews [31]. Given that the focus was on implementation, rather than study outcomes, not all aspects of the PRISMA statement were adopted. Data abstraction involved two stages. First, articles were categorized by reference, study design and methods, participants and setting, study objective, CCM components used, and description of the intervention.

The next stage of data abstraction involved qualitative analysis using the Consolidating Framework for Research Implementation (CFIR), which has five domains: intervention characteristics, outer setting, inner setting, characteristics of the individuals involved, and the process of implementation [26]. It provides a conceptual framework which can be used to understand factors that influence successful implementation in health care, and is based on theories identified by Greenhalgh and colleagues’ widely cited systematic review [26,32]. The CFIR was selected because it includes multiple constructs and theories from peer reviewed studies on evidence-based knowledge dissemination and translation, organizational change and implementation, and uptake of research. It has also been suggested as a framework that can be used to guide the implementation of CCM components in interventions: therefore, it was deemed most appropriate for our study [26]. Table 2 provides summarized descriptions of the CFIR domains.

**Table 2:** **Description of CFIR domains and constructs** [26]

**Domain**	**Definition**
**Intervention characteristics**	The characteristics of the intervention being implemented include whether: the intervention is perceived to be developed external or internal to the organization, there is evidence supporting its effectiveness, and its implementation will be advantageous to its alternatives. Other characteristics include how the intervention is presented, its adaptability, complexity and whether it can be tested on a smaller scale.
**Outer setting**	The external context of the organization includes: patient needs and the ability to meet them, networks with other organizations, pressure to implement the intervention and external policies and incentives to adopt the intervention.
**Inner setting**	Features of the organization including its structural characteristics (such as size, age of the organization and division of labour), networks and communication (such as connections and information sharing between individuals, units and services), cultural norms and values, implementation climate, organizational capacity and readiness for change.
**Characteristics of individuals**	Staff knowledge and belief about the intervention, their ability to execute their respective aspects of the implementation, and their individual stage of change. Other characteristics include individual identification with the organization and other personal attributes.
**Process**	Active change process, the purpose of which is to promote uptake of the intervention by the organization. This is influenced by the level of planning prior to implementation, and engaging organization stakeholders through appointing implementation leaders and champions of the intervention. This includes the ability to execute the implementation of the intervention as planned and to continuously reflect on and evaluate the quality of implementation and intervention as it progresses.

Using qualitative content analysis, implementation barriers and facilitators in 22 articles were mapped on to the CFIR framework. When articles described barriers or facilitators of CCM implementation, they were regarded as “attributive statements”, which were coded under the appropriate constructs and domains. These statements were often found in the discussion and results section of the articles. If the statement was beyond the domains and constructs of the CFIR, then it was still documented. Our approach was modeled after the data abstraction method used in a systematic review by Mair and colleagues [33]. The data abstraction and coding was performed by one reviewer. Interpretative and inductive reasoning were used to map out the attributive statements to the framework.

## Results

Twenty two studies were included in this review. Study descriptions and methodological procedures were summarized in terms of design, measurements, sample size and context, as shown in Table 3. In Table 4 statements reflecting implementation barriers and facilitators from each article were analyzed and coded to their respective domains and constructs under the CFIR framework.

**Table 3:** **Overview of studies on the CCM in primary care**

**Reference/Location**	**Study design, methods**	**Participants (n)/ study setting**	**Objective**	**CCM**	**Intervention**
[34] **Mexico**	Quantitative, pilot study, survey assessing chronic care delivery, and measurement of clinical outcome	Primary care teams (n = 10): physicians, nurses and other professionals were randomly selected and assigned to intervention or control group	Evaluate whether implementation of diabetes quality improvement (QI) project improved patient outcomes	A, B, C, D, E, F	Implementation of QI strategy for diabetes care based on three learning sessions, followed by Plan, Do, Study, Act (PDSA) practice
[35] **USA**	Quantitative, pilot study	Registered nurse, general internists and multi-morbid patients in an urban primary care practice	Assess feasibility of implementing the Guided Care Model	A, C, D, E, F	Guided Care Nurse worked with two physicians to conduct geriatric evaluation, disease management and to coordinate care.
[36] **[USA]**	Quantitative, nonrandomized-prospective clinical trial, survey measuring primary care experiences	Older community patients (n = 150), Registered nurse, general internists (n = 4) in an urban primary care practice	Evaluate intervention to enhance the quality of primary care experiences in chronically ill older persons based on Guided Care model	A, C, D, E, F	Guided Care Nurse provided geriatric assessment, a comprehensive care plan, proactive follow-up, coordination of care, and access to community resources
[19] **[USA]**	Mixed methods study, triangulation of measured clinical processes and outcomes, provider surveys and semi-structured interviews	Team leaders and members (n = 106) in 19 community health centres (CHC)s participating in diabetes QI collaborative	Evaluate whether the Diabetes Health Disparities Collaborative can improve the quality of care in CHCs	A, B, C, D, E, F	CHCs formed QIs teams which attended collaborative learning sessions and adapted QI plans using the PDSA design
[37] **USA**	Quantitative study, self-administered questionnaires on CHC staff	Staff (n = 622) of CHCs (n = 145) participating in QI initiative	Assess predictors of changes in staff morale and burnout at CHCs participating in Health Disparities Collaborative	A, B, C, D, E, F	CHCs participated in quarterly regional or national learning sessions and developed QI teams which utilized the PDSA model
[38] **[USA]**	Quantitative, matched control study, organizational survey, and measurement of care process	CHCs (n = 19) in Health Disparities Cancer Collaboratives, and controls (n = 22) in underserved population	Assess whether CHCs in collaboratives were more likely to implement cancer care process changes	A, B, C, D, E, F	CHCs formed teams to learn how to implement change, facilitated by an expert faculty. Health centers reported and shared QI experiences during monthly teleconferences and three in-person learning sessions
[39] **USA**	Qualitative study, semi-structured interviews, using grounded theory approach	Primary care physicians (n = 24) in multi/single specialty groups or single practices	Examine primary care physicians’ views on obstacles to providing depression care and CCM-based interventions	A, B, C, D, E	Depression screening, structured assessment, patient education, mental healthcare integration, consults and care management
[40] **USA**	Qualitative study, semi-structured interviews, observational notes	Leaders and front-line physicians and nurses (n = 53) in a large multispeciality health group (clinics, n = 5)	Evaluate care changes and processes used to implement CCM	A, B, C, D, E, F	Project leaders and multidisciplinary teams were created to guide implementation, and individual care teams piloted the intervention
[41] **USA**	Quantitative study	Physicians (n = 17) and nurse practitioners (n = 5) in a metropolitan family practice clinic	Describe steps to successfully implement clinic-in-a-clinic diabetes self-management that uses PDSA	A, B, C, D, E, F	Education, behaviour change support, goal setting and follow up provided by nurse practitioner to Type 2 diabetes patients who require more intensive counselling on diabetic self management issues
[42] **USA**	Quantitative, quasi-experimental with concurrent non-randomized controls, measuring intermediate diabetes outcomes	General internists, nurse practitioners, pharmD, clinical health psychologist and nurses in a primary care clinic in a tertiary care academic medical centre	Evaluate intermediate outcome measures of diabetic patients in shared medical appointments (SMA) in comparison to control patients.	A, B, C, D, E	Utilised diabetes registry to identify target patients. Provided decision support by practice guidelines and by including a diabetes specialist in the team. Multidisciplinary team provided didactic group education and individual learning in shared medical appointments
[43] **USA**	Quantitative study, measuring patient participation and changes in diabetes related outcomes	Diabetic patients (n = 275) in a CHC serving low-income Latinos	Assess patient engagement in self management activities and changes in glycosylated hemoglobin (HbA1c).	B	Implementation of diabetes education classes, chronic self-management classes, weekly drop-in sessions, individual counseling, daily exercise classes and bilingual services
[44] **USA**	Qualitative study, structured interview based on ecological systems theory	Team leaders and members of CHCs collaborative (n = 14)	Identify strategies that contributed to CHCs’ successes and challenges in diabetes QI	A, B, C, D, E, F	CHCs assembled teams to participate in the collaborative. They were responsible for coordinating and reporting activities, and electronic registries. The CCM was implemented by a champion panel made of diabetic patients.
[45] **USA**	Qualitative study, telephone interviews	Managers, mental health specialists and care managers in health care organizations (n = 5)	To understand the experiences of project participants in implementing depression improvement model.	A, B, C, D, E	Care management, an improved interface between mental health consultants and primary care clinicians, and preparation of primary care clinicians and practices to provide systematic depression management
[46] **USA**	Quantitative study, measured fidelity to and intensity of CCM implementation	Health care organizations (n = 42) part of QI collaboratives (n = 3)	Measure organizations’ implementation of CCM interventions for chronic care QI	A, B, C, D, E, F	Health care organizations attended three learning sessions together to collaboratively improve performance and focus on implementing small rapid change cycles in their practices
[47] **USA**	Quantitative study	Community based primary care physicians’ offices.	Evaluate the Assessing Care of Vulnerable Elderly Persons (ACOVE) intervention for adults with geriatric conditions	A, B, C, D, E	Case finding, collection of condition-specific clinical data, medical record prompts to encourage performance of essential care processes, patient education and activation, and physician decision support and education
[18] **Canada**	Quantitative study, survey questionnaire evaluating physician normative practices consistent CCM	Physicians (n = 195) in walk-in clinics (n = 29), solo family practices (n = 29), group family practices (n = 104), CHCs (n = 14) and primary care networks (n = 27)	Examine implementation of CCM in different primary care practices	A, B, C, D, E, F	N/A
[48] **USA**	Quantitative study	Diabetic patients (n = 70) over 65 years old in a private medical clinic	Determine whether patients in shared medical appointment meet the American Diabetes Association standards in diabetes self-management education	A, C, D	Implementation of a diabetes self management program using shared medical appointments
[49] **USA**	Quantitative study, questionnaire measuring organization characteristics and care management processes	Administrative leaders of physician organizations (n = 957), including medical groups (n = 621), independent practice associations (n = 336) across the US	Examine the relationship between measures of primary care orientation and the implementation of the CCM	A, B, C, D, F	N/A
[50] **Belgium**	Mixed methods study, CCM implementation survey, analysis of meeting reports	General practitioner (n = 83), dietician (n = 1), pharmacist (n = 46), podiatrist (n = 5) and nurses (n = 90) providing care to type 2 diabetes patients (n = 2300)	Assess degree of implementation of CCM, and facilitators and barriers encountered	A, B, C, D, E, F	Development and implementation of education program for patients on diet or oral therapy, establishment of a local steering group, appointment of program manager, provider education and regional audit
[51] **Canada**	Qualitative study, structured interview with staff	Health administrators, physician leaders, nurses and physicians (n = 12) in a large integrated academic institution.	Examine strategies that promote physician involvement in planning and developing of heart failure care delivery	A, B, C, D, E, F	Detailed analysis of existing heart failure management strategies, a review of best practice strategies and potential future best direction for increased effectiveness
[52] **Netherlands**	Qualitative study, semi-structured interview of project managers	Project directors and managers (n = 16), in health care provider groups (n = 5)	Understand the development, implementation and execution of disease management programs by project leaders and clinicians	A, B, D, E	Implementation of nation-wide disease management program in health organization in the Netherlands
[53] **[USA]**	Qualitative, case study analysis using interviews	Staff and patients from disease-specific shared medical appointments groups (N = 3)	To describe the roles of nurse practitioners in shared medical appointment group visits	A, B, C, D, E, F	Implementation of nurse practitioners in shared medical appointments

Quality improvement; QI, Chronic Care Model; CCM, Plan Do Study Act model; PDSA, Guided Care Nurse; GCN, Community Health Center; CHC; N/A; not available.

CCM components.

A = Delivery system redesign.

B = Self management support.

C = Decision support.

D = Clinical information system.

E = Health system organization.

F = Community linkages.

**Table 4:** **Thematic analysis shows the barriers and facilitators identified by the studies mapped on to their corresponding CFIR domains and constructs**

**Construct**	**Domain**	**Facilitator [reference number]**	**Barrier [reference number]**
	**1. Intervention characteristic**		
**A. Intervention source**			
**B. Evidence strength & quality**			“Limited guidance on prepared practice team development” [40]
**C. Relative advantage**		“Patient screened by staff before seeing physician ” [39], “Structured assessment in patient education” [39]	
**D. Adapability**		“Integrating Guided Care nurse in work flow” [36], “Processes integrated in to existing clinical operations” [43], “CCM adaption within context of daily practice” [48], “Program tailored to region needs” [50], “Adapting communication system to local context” [52], “Integrated project to routine care” [52]	
**E. Trialability**			
**F. Complexity**			“Intervention was too complex, targeted different components resulting in many priorities” [50]
**G. Design quality & packaging**		“Nurse training for components of intervention” [35], “Curriculum should be specific to CCM intervention” [36], “Different intervention model options were offered” [19], “Structured learning sessions and support by health collaborative” [44], “Guideline development” [50]	“Intervention was too disease specific and did not address chronic care principles” [45]
**H. Cost**		“Low-cost program relied on community health workers, mentors and non-clinical staff” [43], “Financially viable” [48], “Sufficient funding” [37]	
	**2. Outer setting**		
**A. Patient needs & resources**		“Community health workers important in addressing patient needs” [43], “Program accessible and offered peer support” [43]	“Need for patient resources” [19], “Patients uninsured or Medicare insured” [38], “Language barriers” [38], “Language and literacy issues” [44]
**B. Cosmopolitanism**			
**C. Peer pressure**			
**D. External policies & incentives**			“Poor organization of primary care in region” [50]
	**3. Inner setting**		
**A. Structural characteristics**		“Development of prepared practice teams” [40], “Electronic medical record (EMR) implementation and clinic remodelling” [39], “Recruitment of multilingual staff and interpreters to address language barriers” [44], “Worked with human resources to change organizational policies” [44], “Role of specialist in supporting and supervising other staff” [45], “Addition of technology system” [52], “Nurse practitioner role in implementation” [53]	“Staff turnover” [19], “Large size of medical group” [40], “Unions unsupportive of staff role change” [40], “Medical director turnover” [38], “Need to expand role of provider” [44], “Staff turnover and loss meant very few staff could assume additional responsibilities” [44], “Lack of staff expertise in team approach to implementation” [48], “Lack of flexibility in reorganizing model of care” [52], “Smaller organizations had difficulty addressing barriers” [45]
**C. Culture**		“Support from primary care physicians” [35], “Support from physicians” [36], “Recognition of benefit of care managers” [39], “Stable work relationships” [40], “Recognition of patient role in self management” [44], “Persistence despite extra work” [44], “Organizational culture and enthusiasm for care improvement” [45], “Promoting multidisciplinary approach” [51], “Change to patient-centred care” [52], “Receiving personal recognition” [37]	“Providers need for clear structure and autonomy” [19], “Organizational culture unsupportive of change” [40], “Lack of commitment or tradition of working in interdisciplinary teams” [50], “Difficulty changing provider care to patient-centered care” [52], “Rigid role expectations and thought processes” [52]
**D. Implementation climate**		“Clear, shared long term commitment for change” [40], “Recognized need for change” [40], “Work credit to ensure staff buy-in” [42], “Institutional commitment for change” [45], “Commitment to follow guidelines” [48], “Provider dissatisfaction with current system” [50], “Financial reimbursement for attending meetings” [51], “Organizational will to promote change and manage change” [51] “Career promotion opportunities” [37], “Incentives such as skill development” [37]	“Lack of physician interest in addressing communication barriers with specialists” [39], “Disagreement on need for standardized care” [40], “Lack of commitment and interest by chief physician” [40], “Lack of committed vision” [45], “Difficult to motivate providers due to program uncertainty” [50], “Lack of provider commitment” [50]
**E. Readiness for implementation**		1. “Used pre-existing available resources: information system and education program” [34], “Buy-in from senior management” [19], “Previous implementation of structured assessment in EMR” [39], “Importance of project leaders” [52], “Sufficient staff personnel” [37]	“Low staff and space resources” [43], “Lack of reimbursement strategy” [45], “Lack of financial resources” [50], “Software builder did not meet goals” [52], “Limited financial resource” [34], “Hidden and unexpected implementation expenditures” [52]
	**4. Individual characteristics**		
**A. Knowledge & beliefs about intervention**		“Increase awareness and education about program to providers” [41], “Observation of program processes by providers” [42], “Patient registry received interest in providers” [44], “Clinical assessment tool accepted and endorsed” [45], “Information campaign to increase awareness and knowledge” [50], “Education about project goals & process” [51], “Demonstration of project benefit to physicians” [51], “Staff morale and burnout reduction associated with reports of improved care outcomes” [37]	“Needed more information on structured assessment” [39], “Unconvinced of usefulness of structured assessment for diagnoses” [39], “Lack of program information from providers that were not full time” [41], “Physician buy-in and adoption of intervention was not uniform” [47], “Fear of losing patient control to education program” [50], “Time needed for provider trust in program” [50], “Clinicians sensitive to workload and time commitment” [45]
**B. Self-efficacy**			“Fatigue and apathy from pace of change” [40], “Decreased staff participation in intervention results in low morale” [37]
**C. Individual identification with organization**			
**D. Personal attributes**			
	**5. Process**		
**A. Planning**		“Realistic expectations for measureable results” [40], “Consultation with focus groups for change process priorities” [50], “Physician involvement in planning” [51], “Utilized patient and physician experience in project development” [51], “Goals of QI as drivers of planning ” [52]	“Lack of details on care change goals & outcomes” [40], “Too many priorities and uncoordinated change processes” [40],“Need for stronger program goals delineation” [41], “Lack of clear program aim at the start of campaign” [50]
**B. Engaging**		“Supportive administration and intervention champion” [19], “Strong physician leadership” [40], “Supervisor support” [40],“Strong registered nurse leadership” [40], “Clear goals by leaders” [40], “Strong supportive leader” [45], “Commitment & support of senior leaders” [50], “Recruitment of physician champion” [51], “Engaging champions with physicians” [51], “Presence of strong champion” [37]	“Need for more senior management support” [19], “Need for intervention champion” [19], “Lack of accountability by leadership” [40], “Leaders face multiple uncertainties and distractions” [40], “Champion provider had limited time with patients” [44], “Change difficult without leadership endorsement” [44], “Lack of active provider champion” [44]
**C. Executing**		“Coordination of program components” [41], “Target screening of at risk patients” [39], “Pre-visit screening by staff before seeing physicians” [39], “Pre-visit by nurse and clerical staff” [40], “Approached patient as a team” [44], “Health care organizations part of collaborative had high CCM fidelity and moderate intensity” [46], “Flexible meeting times and locations” [51], “Fair distribution of tasks” [37]	“Inadequate time to work on intervention” [19], “Difficulty with patient registry” [19], “Need for technical support” [19], “Competing demand of simultaneous EMR implementation” [40], “Physicians not engaged in change processes” [40], “Patient registry lacked IT support” [44], “Difficult to implement all CCM elements at high intensity in 12 months” [46], “Screening all patients time was consuming” [39], “Time constraints in appropriate assessment” [48], “Buy-in from staff not sufficient to sustain program” [48], “Increase in administrative burden” [50], “Patient involvement in own care was difficult” [52]
**D. Reflecting & evaluating**		“Periodic reviews and feedback of performance” [36], “Staff provided feedback on process design” [41], “Continuous assessment and revisions of program” [41], “Support from monthly feedback and learning sessions” [44]	“Insufficient time to measure change” [40], “Lack of useful measure of change” [40], “Lack of EMR and billing codes were barriers for measurement of processes and outcomes” [48], “Implementing and measurement was labour intensive” [48]

### Facilitators

#### Networks and communication

Strong networks and increased communication between health care providers and organizations were fostered by collaboration across disciplines and specializations during care change processes [39,40,44,50,51]. Communication was reportedly supported by regular group meetings with supervisors and managers to discuss implementation issues, computerized information sharing and clinical assessment tools [41,45,52].

#### Culture

An organizational culture that promotes multidisciplinary, or patient centered care, was identified as important during implementation [45,51,52]. Support from clinical providers and the recognition of their importance in care change efforts was found to increase uptake of the CCM in primary care [35,37,39].

#### Implementation climate

Studies found that implementation climate was influenced by commitment and recognition for the need for change from the organization [40,45]. Willingness to advance and manage change was evident through incentivizing provider buy-in using financial reimbursement and work credit for project involvement [37,42,51].

#### Structural characteristics

Operationalization of CCM components was facilitated by health care providers, particularly specialists and non-physician staff such as nurse practitioners, who had to expand their responsibilities and scope of practice [45,53]. This sometimes required changing organizational policies and development of care teams to meet implementation needs [40,44].

#### Engaging

Strong, committed and engaging leadership in the form of supportive administration and supervisors, with clear goals, was cited as a facilitator [40,45,50]. This included the appointment of an intervention champion to promote uptake of the model within the organizations [19,37,51]. Leadership roles were not limited to physicians, other health care providers such as nurse practitioners were found to play a major role in implementation [40].

#### Knowledge and beliefs about the intervention

Provider knowledge about CCM interventions was promoted through observing the execution process by other staff and education about project goals [42,50,51]. Strategies used to foster beliefs of the CCM effectiveness in care providers, particularly physicians, included demonstration of its benefits to their practice and sharing reports of patient improvements [37,51].

### Barriers

#### Executing

Many studies identified barriers related to executing intervention processes. Implementing the multiple components of CCM into practice created additional responsibilities for staff who were limited by time constraints [19,40,48,50]. Pearson & colleagues found that operationalizing the model elements at a high level of intensity, within a short time frame to be challenging [46]. Sustainability of the intervention was found to be difficult in some studies; in some instances, staff buy-in, an important aspect of implementation, was not enough to ensure program longevity [48].

#### Structural characteristics

Characteristics of the healthcare organization such as its size, whether it adopted a team-based approach and its flexibility in reorganizing care, were seen to influence the success of CCM adoption [40,45,48,52]. Institutional factors such as staff turnover and loss meant increased burden of responsibilities on existing providers [19,44] 10). Leadership turnover, particularly that of a medical director, was cited as a barrier towards implementing care change processes [38].

#### Readiness for implementation

Organizational readiness for the CCM was found to be impacted by the lack of interest and commitment from leadership and unavailability of resources for implementation [40,45]. Lack of resources that influenced readiness included low funding, lack of provider reimbursement strategies and low staff numbers [34,43,45,50].

#### Engaging

Many studies found that execution of the intervention processes was challenging without support and accountability from senior leadership [19,20,44]. Without the presence of an intervention champion, endorsement of the CCM initiative was found to be limited in healthcare providers [19].

#### Knowledge and beliefs

Provider buy-in was greatly influenced by knowledge and beliefs about the intervention, particularly if they had misconceptions, were unconvinced of its effectiveness or lacked information [39,47,50]. Acceptance of the interventions by clinicians required time, and was also affected by the workload associated with implementing and executing the intervention components [45,50].

## Discussion

This review identified multiple barriers and facilitators of implementing the CCM across various primary care settings. The major emerging themes were those related to the *inner setting* of the organization, the *process* of implementation and *characteristics of the individual* healthcare providers. These included: culture of the organization, its structural characteristics, networks and communication, implementation climate and readiness, supportive leadership, and provider attitudes and beliefs.

Every primary care organization possesses its own cultural norms, practices and leadership. It is impossible to achieve change without adopting an approach that considers the individual and the team of providers, the organization setting and the greater system within which it is embedded [54]. Wolfson and colleagues attributed the success of QI in different primary care practices to facilitators in various levels of the organization including: presence of an initiative champion; physician, staff and patient cooperation; leadership investment; team practice and progress tracking [55]. The uptake of CCM elements in the studies required a primary care culture supporting willingness to change and quality improvement at the individual clinician, team and organizational levels. Implementation was most successful when there was a shared vision and a recognized need across the organization for new care change approaches to promote effective execution of the CCM [35,36,39,44,52].

Transforming care practices in an organization requires a supportive culture of change and learning [23]. Clinical provider beliefs and attitudes about evidence-based practice can influence the culture and learning environment, particularly when the provider perceives the evidence as unreflective of their day-to-day clinical decision making. This suggests the need to involve clinicians in early stages of intervention development and implementation [22]. Interventions that incorporated providers, patients and their experiences in the planning phase of the intervention were more successful in operationalizing the CCM [50,51]. This approach may bridge the cultural divide between leadership and clinical providers, which can hinder quality improvement efforts if left unaddressed. On the other hand, literature shows that lack of a group-oriented culture, as well as hierarchical relationships where the leadership is unsupportive of change, are negatively associated with implementation of care change processes [55]. Marshall and colleagues highlight the importance of culture and cultural change when implementing clinical governance in primary care. Cultural traits that support implementation efforts include commitment to accountability by the organization, willingness for collaborative work and learning, and ability to evaluate and reflect on mistakes [56].

Implementing and managing change processes in primary care can require time and flexibility. Organizational transformation can be slow and resistant to change, while spread of best-practice can be a challenge [57]. In some cases, even when an organization’s culture is supportive of the CCM, the inner structures of the primary care organization, such as a lack of staff and financial resources or a lack of clinician expertise, can impede organizational readiness for implementation and cause unexpected setbacks [34,48,52]. A study evaluating the implementation of evidence-based practice revealed that the current primary care system is not adaptive to rapid change, or accommodating to the additional duties associated with adopting new interventions. What this suggests is the need to set realistic implementation goals that are reflective of the organization and staff capacity for changes associated with the CCM. This requires comprehensive planning at all stages of component adaptation, to mitigate impeding factors such as rigid bureaucracies and organizational policies.

On the other hand, clinical leaders and champions can be drivers of change by ensuring the availability of resources and providing adequate staff supports [58]. Indeed, leadership support for change has been shown to be positively associated with QI outcomes and sustainability in primary care [24].

Implementation of CCM in primary care requires tailoring interventions to the local context, as well as altering the context, for the process to be successful. The two can co-adapt and evolve during the implementation process, thereby ensuring sustainability [59]. The majority of the studies included in the review identified the impact of the CCM on existing routines, practices, and culture of the organization. There was variability in how each organization adapted the CCM, i.e., translating the framework’s components into practice resulted in implementation heterogeneity. What became clear is that a standardized one-size-fits-all approach was difficult to put into practice when the components were conceptualized differently by each primary care organization.

Tailoring the intervention necessitates accounting for innovation-promoting and hindering factors at different levels of the organization, as well as reconfiguring aspects of the primary care setting itself [50].

This systematic review has several limitations. First, our search strategy meant that we did not assess: grey literature, studies that have not been published in peer-reviewed journals or those published in languages other than English; therefore, articles that were relevant to our review may not have been included. The search may have excluded studies that implemented CCM-based interventions but which were not explicitly referred to as such in the articles. In addition to the challenge of consistently identifying such studies, it would be difficult to be certain that implementation issues were reflective of issues relevant to the CCM. Another limitation is that the articles that were included were selected and assessed by one reviewer, thus limiting the reliability of the selected studies. Given that the articles were abstracted qualitatively by a single data abstractor, there is a possibility of bias in how the attributive statements were mapped under CFIR constructs and domains. While abstraction and coding was carried out by one reviewer, extensive and continuous discussion took place between both authors occurred during the study selection and data abstraction process. While using the CFIR as a guiding framework is a strength of our review, the numerous construct and sub-constructs meant that areas with few facilitators and barriers identified received less consideration (although these were captured in Table 4).

## Conclusion

These findings highlight the need to evaluate factors that influence successful implementation of CCM in primary care. The CFIR can be used to guide the formative evaluation processes of CCM interventions. Assessment of organizational capacity and needs is important prior to and during the implementation of the intervention, in order to gain a better understanding of health care providers and organizational perspective. The barriers and facilitators identified under the CIFR domains can be used to build knowledge on how to adapt the CCM to different primary care settings.

## Abbreviations

QI: Quality improvement
CCM: Chronic Care Model
CFIR: Consolidating Framework for Research Implementation
PDSA: Plan Do Study Act model
GCN: Guided Care Nurse
CHC: Community Health Center
EMR: Electronic Medical Records
